# The common motives for appendectomy in Hail Region Saudi Arabia

**DOI:** 10.3934/publichealth.2020011

**Published:** 2020-03-03

**Authors:** Fawaz D Alshammari, Hanan A Oreiby, Hussain Gadelkarim Ahmed, Khalid Alshaghdali, Jerold C Alcantara, Gamal Mohamed Elawad Ahmed, Sara A Seifeldin, Emad Abboh Abdallah Abboh, Waleed Mansi Al Shammari, Fawzia Mutasim M Al Tayeeb, Bandar S Al Saif, Ali Ahmed Al Qahtani, Samir Abdulkarim Alharbi, Ibtihag Siddig Elnaem

**Affiliations:** 1 Department of Clinical Laboratory, College of Applied Medical Sciences, University of Hail, Hail, Saudi Arabia; 2 Consultant, Histopathology, King Khalid Hail Hospital, Hail, Saudi Arabia; 3 College of Medicine, University of Hail, Hail, Saudi Arabia; 4 Department of Histopathology and Cytology, FMLS, University of Khartoum, Sudan; 5 King Khalid Hail Hospital, Clinical Pathology Department, Hail, Saudi Arabia; 6 College of Applied Medical Sciences, Department of Clinical Laboratory Science, Shaqra University, Saudi Arabia; 7 College of Dentistry, University of Hail, Saudi Arabia

**Keywords:** appendix, appendicitis, lymphoid hyperplasia, Saudi Arabia

## Abstract

**Background:**

Appendectomy remains the most common emergency surgery. With the lack of literature from Saudi Arabia regarding the treatment for appendix disorders, this study aimed to identify the common motives for appendectomy in Northern Saudi Arabia.

**Methodology:**

Data referring to be resected appendix patients who were diagnosed during the period from January 2018 to December 2018 were included in the present study. The diagnosis of the resected appendix was confirmed by conventional histopathology.

**Results:**

The most common cause for the appendectomy was acute appendicitis followed by gangrenous perforated appendicitis, chronic appendicitis, and lymphoid hyperplasia, representing 85/129(66%), 33/129(26%), 8/129(6%), and 3/129(2%), in this order.

**Conclusion:**

Appendectomy is a common procedure for the treatment of a large section of patients with appendicitis and appendicitis like clinical features. Acute appendicitis was the most motive for appendectomy followed by gangrenous perforated appendicitis in Northern Saudi Arabia.

## 1. Introduction

Appendicitis is the most commonly performed emergency abdominal surgery. The appendix can also be the site of a variety of neoplasms and unusual inflammatory conditions [1]. Acute appendicitis is the most common reason for abdominal surgery in children. Luminal obstruction of the appendix progresses to suppurative inflammation and perforation, which causes generalized peritonitis or an appendix mass/abscess. Classical features include periumbilical pain that migrates to the right iliac fossa, anorexia, fever, and tenderness and guarding in the right iliac fossa. Atypical presentations are particularly common in preschool children. A clinical diagnosis is possible in most cases, after a period of active observation if necessary; inflammatory markers and an ultrasound scan are useful investigations when the diagnosis is uncertain [2].

The correlation between clinical and histopathology findings in appendicitis has been highlighted by many studies. However, the impact of this correlation on the surgical decision to remove a normal-looking appendix is still vague, with no clear definition of positive appendicitis [3]. Incidental appendectomy can be defined as the removal of a clinically normal appendix during another surgical procedure unrelated to appendicitis or other appendicular diseases. Careful inspection of the entire abdominal cavity is useful for patients undergoing major abdominal surgery such as donor hepatectomy [4].

Histopathological examination of the appendix is routinely performed [5]. The findings of abnormal pathologies on histopathological examination of the appendix which could potentially impact on the management of the patients justify the current practice of routine histopathological examination of the resected appendix. However, there paucity of data from different regions of Saudi Arabia regarding reasons of appendectomy and whether surgical intervention is deemed necessary or not. Also comparing the causes of appendectomy in Saudi Arabia and global reasons may give some influence for the better management of appendicitis in Saudi Arabia. Therefore, this study aimed to identify the common motives for appendectomy in Northern Saudi Arabia.

## 2. Materials and methods

In the current study data regarding histopathological diagnosed resected appendix were retrieved from the Department of Pathology at King Khalid Hospital, Hail, Northern Saudi Arabia. Data referring to resected appendix patients who were diagnosed during the period from January 2018 to December 2018 were included. The diagnosis of the resected appendix was confirmed by conventional histopathology.

The histological examination of resected appendix specimens was completed to realize the valuation process role, by giving a pathology category classification (Inflammation, malignant and benign lesions).

### 2.1. Statistical analysis

Data analysis was done by using the Statistical Package for Social Sciences (SPSS version 16; SPSS Inc, Chicago, IL). SPSS was used for analysis of data to calculate the frequencies and percentages of different variables.

### 2.2. Ethical consent

Our study protocol was confirmed according to the 2013 Declaration of Helsinki and this study was approved by the ethics committee of the College of Medicine, University of Hail, Saudi Arabia.

## 3. Results

**Figure 1.**
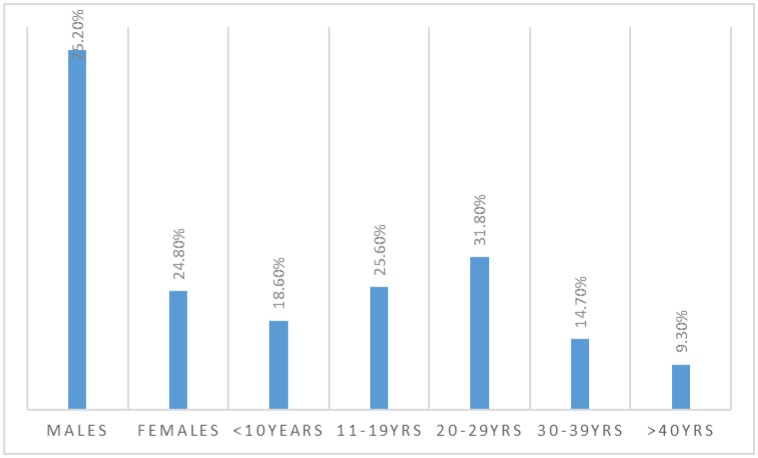
Description of the study subjects by sex and age.

This study examined 129 patients underwent appendectomy, their ages ranging from 4 to 56 years. Out of 129 patients, 97/129(75.2%) were males and 32/29(24.8%) were females giving males, females' ration of 3.03 to 1.00. Most of the patients were found at the age group 20–29 years followed by 11–19 and < 10 years, representing 41, 33 and 24 patients as indicated in Figure 1 and Table 1.

**Figure 2.**
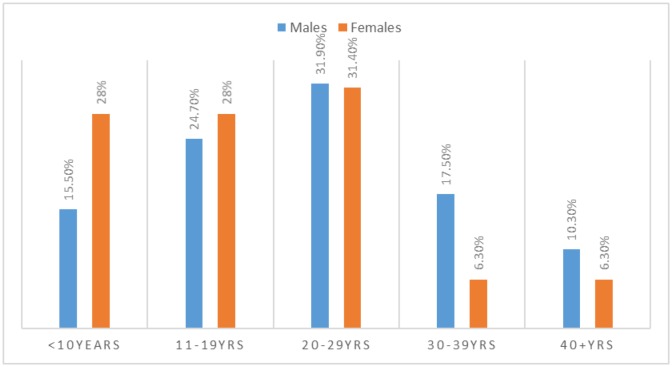
Description of patients by age and sex.

As indicated in Table 1, Figure 2, most males were aggregating at the age group 20–29 years followed by 11–19 and 30–39 years constituting 31, 24, and 17 respectively, whereas, most females were gathered at the age range 20–29 years followed by both 11–19 & < 10 years representing 10, and 9 patients, in this order.

**Table 1.** Distribution of patients by age and sex.

Age group	Males	Females	Total
< 10 years	15	9	24
11–19	24	9	33
20–29	31	10	41
30–39	17	2	19
40 +	10	2	12
Total	97	32	129

**Figure 3.**
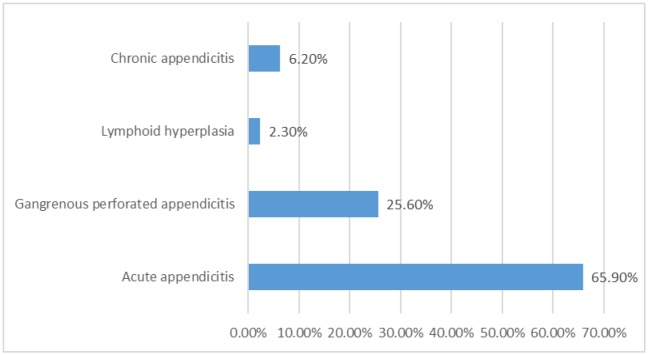
Description of appendectomy by pathology.

As shown in Figure 3, photomicrograph 1 and 2, the most common cause for the appendectomy was acute appendicitis followed by gangrenous perforated appendicitis, chronic appendicitis, and lymphoid hyperplasia, representing 85/129(66%), 33/129(26%), 8/129(6%), and 3/129(2%), in this order.

**Photomicrograph 1.**
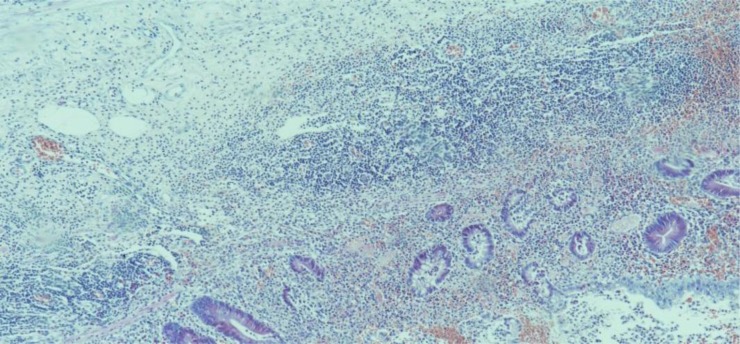
Acute appendicitis. H&E, X100.

**Photomicrograph 2.**
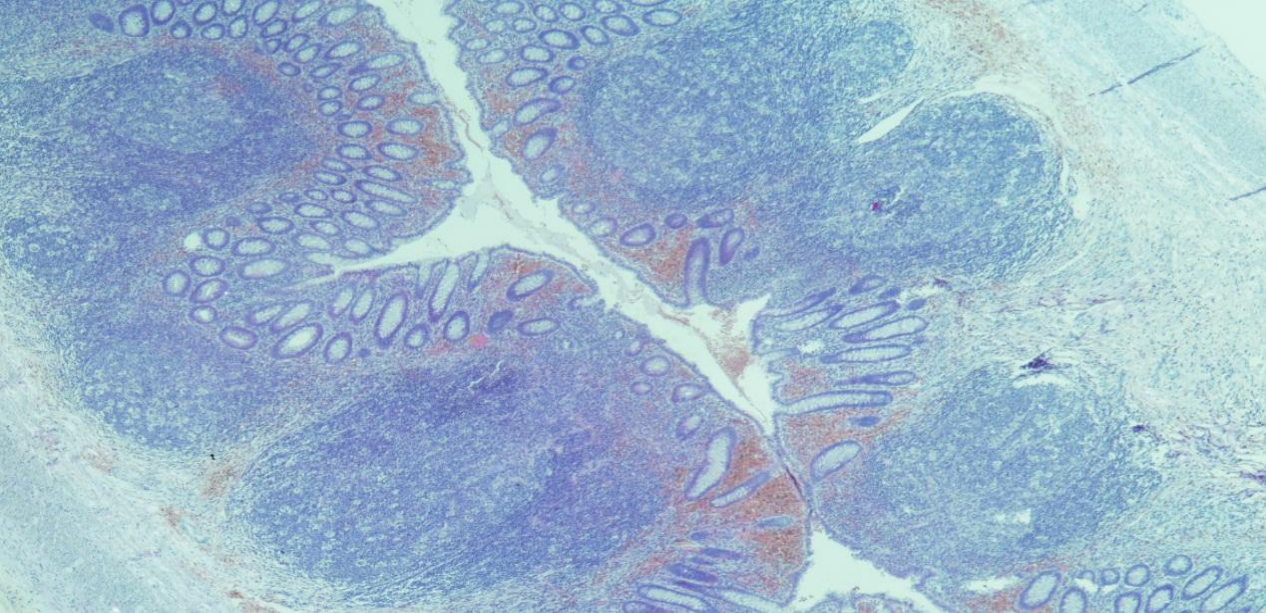
Acute on chronic appendicitis. H&E, X100.

**Table 2.** Description of appendectomy by pathology and sex.

Diagnosis	Males	Females	Total
Acute appendicitis	69	16	85
Gangrenous perforated appendicitis	22	11	33
Lymphoid hyperplasia	1	2	3
Chronic appendicitis	5	3	8
Total	97	32	129

**Figure 4.**
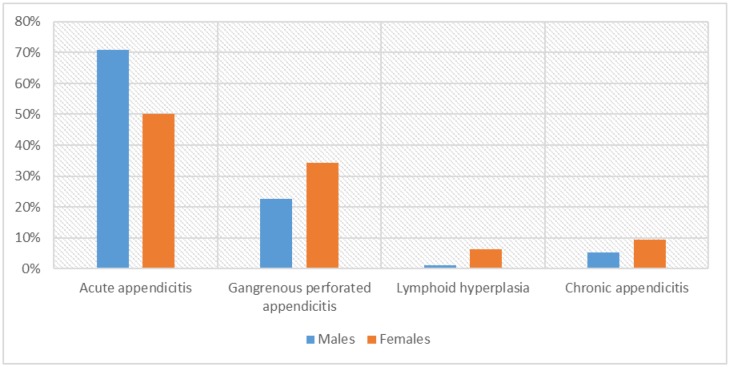
Appendectomy by pathology and sex.

As indicated in Table 2, Figure 4, acute appendicitis was identified in 69/97(71%) of the males and 16/32(50%) of the females. Gangrenous perforated appendicitis was identified in 22/97(22.7%) of the males and 11/32(34.4%) of the females.

Lymphoid hyperplasia was identified in 1/97(1%) of the males and 2/32(6.3%) of the females.

Chronic appendicitis was identified in 5/97(5.2%) of the males and 3/32(9.4%) of the females.

**Figure 5.**
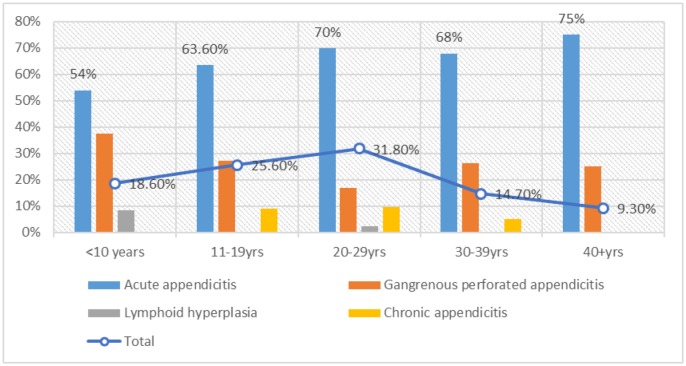
Description of the proportions of pathology within each age group.

As summarized in Table 3, Figure 5, acute appendicitis was predominantly observed at the age group 20–29 years followed by 11–19 years, representing 29/85(34%) and 21/85(24.7%) correspondingly. Gangrenous perforated appendicitis was greatly seen at the younger age ranges < 10 years & 11–19 both representing 9/33(27.3%). Chronic appendicitis was increasingly seen in the age group 20–29 years followed by 11–19 years constituting 4/8(50%) and 3/8(37.5%).

**Table 3.** Description of appendectomy by pathology and age.

Diagnosis	< 10 years	11–19	20–29	30–39	40 +	Total
Acute appendicitis	13	21	29	13	9	85
Gangrenous perforated appendicitis	9	9	7	5	3	33
Lymphoid hyperplasia	2	0	1	0	0	3
Chronic appendicitis	0	3	4	1	0	8
Total	24	33	41	19	12	129

## 4. Discussion

The appendectomy is one of the frequent emergency surgical procedures in many countries. The precise clinical assessment is an important step for the determination of subsequent appendectomy. The present study was aiming at assessing the diagnostic findings after resecting of the appendix.

The majority of patients in this series were males. Although there is a variation in the incidence rates in different population settings, some studies have shown the predominant of males in relation to appendectomy [6],[7]. The majority of the cases in this study were cases of acute appendicitis followed by gangrenous perforated appendicitis. Appendicitis is the most common abdominal surgical emergency. The reported lifetime risk of appendicitis in the United States is 8.6% in men and 6.7% in women, with an annual incidence of 9.38 per 100,000 persons [8]. These figures showing an increased incidence among men compared to females, which is in line with our findings in the present study. Many studies have investigated the association between time interval and the incidence of complicated appendicitis. Complicated appendicitis incidence was associated with overall elapsed time from symptom onset to admission or operation; short appendectomy in-hospital delay did not increase the risk of complicated appendicitis. Prompt surgical intervention is warranted to avoid additional morbidity, enabling quicker recovery in this population [9]. Progression of the inflammatory process can lead to abscess, ileus, peritonitis, or death if untreated. Complicated appendicitis refers to the presence of gangrene or perforation of the appendix. Free perforation into the peritoneal cavity can lead to purulent or feculent peritonitis. A contained perforation can lead to appendix abscess or phlegmon (inflammatory mass) [8].

However, there is a lack of literature regarding appendicitis from Saudi Arabia. During our literature search we come across a study reviewed histopathological records of 480 resected appendices submitted to histopathology department at Arar Central Hospital in the Northern Border Province of Saudi Arabia over the period of 3 years from July 2011 to June 2014 were reviewed retrospectively, to determine acute appendicitis, complication (gangrene, perforation) rate, negative appendectomy rate, histopathological diagnosis and unusual finding on histology. Out of 480 specimens of the appendix, appendicitis accounted for 466 (97.0%) with peak occurrence in the age group of 11 to 50 years in male and 11 to 40 years in the female. Histopathological diagnosis includes acute appendicitis 250 (52.0%), suppurative appendicitis 135 (28.0%) acute gangrenous appendicitis 60 (12.5%), perforated appendicitis 9 (2.0%), chronic appendicitis 12 (2.5%) [10]. Although, some of these findings were relatively similar to findings but some showing some sort of discrepancies, which might be attributed to the sample size variation.

In the present study lymphoid hyperplasia was identified in 2% of the patients. Hyperplasia of the lymphoid tissue in the mucosa or submucosa has been suggested as the most frequent process leading obstruction of the appendix lumen. It may appear as acute catarrhal appendicitis, with increasing production of symptoms. Lymphoid hyperplasia may be caused by infections or by inflammation, such as in inflammatory bowel disease [8].

Acute appendicitis was commonly seen among 20–29 years' age group followed by 11–19 years, whereas, gangrenous perforated appendicitis was observed more frequently among younger population < 20 years. Appendicitis before age 20 years has been shown to impact the risk of many inflammatory disorders, perhaps through underlying immunological mechanisms [11]. In spite of being a relatively common complaint, the diagnosis of appendicitis in children can ascertain to be challenging in many cases [12]. Nevertheless, such findings regarding the relationship between age and the clinical stages of appendicitis were previously reported from Saudi Arabia showing relatively similar findings [10]. However, the findings of the present study may be useful comparative template for the reasons of appendectomy in neighboring countries and global, which may inspire further investigations in order to provide guidelines for better appendix disorders management, as well as, reducing unnecessary appendectomy.

Though the present study provided important information about reasons for appendectomy with a paucity of such data from Saudi Arabia, it has some limitations including its retrospective setting, which lack some clinical data correlations.

## 5. Conclusion

Appendectomy is a common procedure for the treatment of a large section of patients with appendicitis and appendicitis like clinical features. Acute appendicitis was the most motive for appendectomy followed by gangrenous perforated appendicitis in Northern Saudi Arabia.
